# PRMT1 promotes mitosis of cancer cells through arginine methylation of INCENP

**DOI:** 10.18632/oncotarget.6050

**Published:** 2015-10-09

**Authors:** Xiaolan Deng, Gottfried Von Keudell, Takehiro Suzuki, Naoshi Dohmae, Makoto Nakakido, Lianhua Piao, Yuichiro Yoshioka, Yusuke Nakamura, Ryuji Hamamoto

**Affiliations:** ^1^ Section of Hematology/Oncology, Department of Medicine, The University of Chicago, Chicago, IL, USA; ^2^ Department of Pharmaceutical Analysis, School of Pharmacy, China Medical University, Shenyang, P. R. China; ^3^ Biomolecular Characterization Unit, RIKEN Center for Sustainable Resource Science, Wako, Saitama, Japan

**Keywords:** INCENP, Aurora kinase B, PRMT1, arginine methylation

## Abstract

Inner centromere protein (INCENP) is a part of a protein complex known as the chromosomal passenger complex (CPC) that is essential for correcting non-bipolar chromosome attachments and for cytokinesis. We here demonstrate that a protein arginine methyltransferase PRMT1, which are overexpressed in various types of cancer including lung and bladder cancer, methylates arginine 887 in an Aurora Kinase B (AURKB)-binding region of INCENP both *in vitro* and *in vivo*. R887-substituted INCENP revealed lower binding-affinity to AURKB than wild-type INCENP in the presence of PRMT1. Knockdown of PRMT1 as well as overexpression of methylation-inactive INCENP attenuated the AURKB activity in cancer cells, and resulted in abnormal chromosomal alignment and segregation. Furthermore, introduction of methylation-inactive INCENP into cancer cells reduced the growth rate, compared with those introduced wild-type INCENP or Mock. Our data unveils a novel mechanism of PRMT1-mediated CPC regulation through methylation of INCENP.

## INTRODUCTION

During mitosis, the chromosomal passenger complex (CPC) comprising inner centromere protein (INCENP), Aurora Kinase B (AURKB), Borealin/Dasra B and Survivin plays critical roles at the centromere in ensuring strict chromosome alignment and segregation [1, 2]. It is known that inappropriate chromosomal segregation and cytokinesis due to the deregulation of CPC member proteins leads to aneuploidy and cancer development [3]. INCENP is the first-identified passenger protein [4] and forms a complex with AURKB, which is essential for proper cell division [5, 6]. The INCENP N-terminus is required for CPC localization to centromeres [7], and this region forms a three-helix bundle with N-terminus of Borealin and C-terminus of Survivin, both of which contribute to centromere targeting of CPC [1, 8]. The C-terminus of INCENP binds AURKB through its highly-conserved IN box and this binding is essential for triggering AURKB activation [9]. In fact, this interaction is shown to enable AURKB to phosphorylate a C-terminal TSS (threonine-serine-serine) motif in INCENP and threonine 232 in the T-loop of its kinase domain, which results in full activation of AURKB [10–12]. Hence, the interaction between INCENP and AURKB is considered to be critical for the full activation of AURKB kinase activation and the CPC function during mitosis. In addition to phosphorylation of INCENP by AURKB, phosphorylation of threonine 59 and threonine 388 on INCENP by cyclin-dependent kinase 1 (CDK1) is also known to be necessary for the recruitment of polo-like kinase 1 (PLK1) to the kinetochore [13]. However, other post-translational modifications including methylation on INCENP have not been characterized.

Protein arginine methyltransferase 1 (PRMT1) is a type I arginine methyltranferase, which produces mono-methylarginine (MMA) and assymetric di-methylarginine (ADMA), and catalyze methylation of the third arginine of histone H4 [14, 15]. We previously reported that PRMT1 was overexpressed in various types of cancer including lung and bladder cancers, and knockdown of PRMT1 resulted in the growth suppression of cancer cells [16].

In the present study, we demonstrate that PRMT1 methylates arginine 887 on INCENP and this methylation plays an important role in the interaction between INCENP and AURKB. This is a novel regulatory mechanism of the CPC function that is associated with human carcinogenesis through arginine methylation of the non-histone protein.

## RESULTS

### PRMT1 methylates arginine 887 on INCENP both *in vitro* and *in vivo*

**Figure 1:**
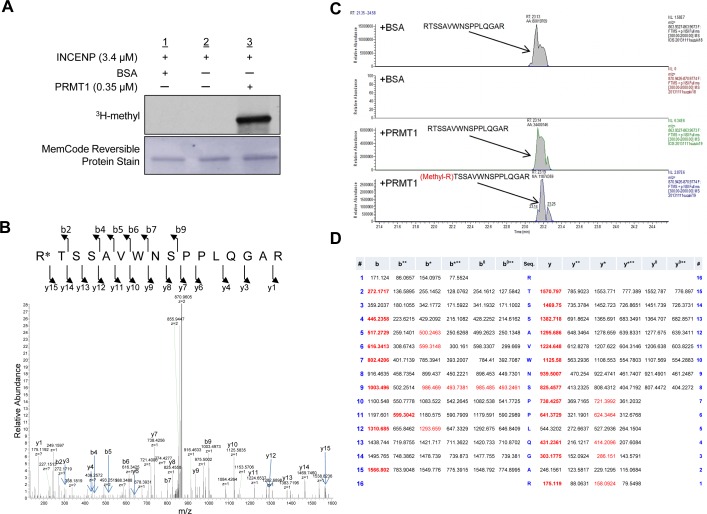
PRMT1 methylates INCENP *in vitro* **A.** Recombinant INCENP protein was methylated by PRMT1. An *in vitro* methyltransferase assay was performed by using purified GST-tagged INCENP and PRMT1 recombinant proteins. Methylated INCENP was detected by fluorography. Amounts of loading proteins were evaluated by staining with MemCode^TM^ Reversible Protein Stain (Thermo Fisher Scientific). **B.** The LC-MS/MS spectrum corresponding to the mono-methylated INCENP 887–902 peptide of a tryptic digest of which *in vitro* methylated INCENP by PRMT1. The 14 Da increase of the Arg 887 residue was observed by y15 ion. **C.** Selected full MS ion chromatograms of unmodified and mono-methylated INCENP 887-902 peptides in the LC-MS/MS. **D.** The theoretical values of MS/MS fragments ions of the Arg 887 mono-methylated INCENP 887–902 peptides are summarized in the table. The abbrevations of fragment ion types were indicated by the MASCOT program (http://www.matrixscience.com/help/fragmentation_help.html). The observed ions in Figure 1B were indicated in red letters.

**Figure 2:**
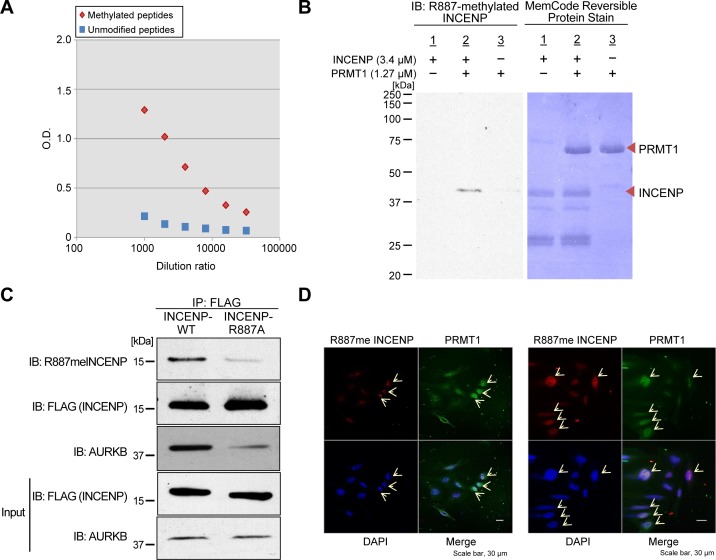
*In vivo* methylation of INCENP by PRMT1 **A.** Determination of the titer and specificity of the anti-mono-methylated R887 INCENP antibody analyzed by ELISA. **B.** Validation of the anti-mono-methylated R887 INCENP antibody. Recombinant GST-tagged INCENP protein and S-adenosyl-L-methionine (SAM) were incubated in the presence of BSA or recombinant PRMT1. Samples were immunoblotted with the anti-mono-methylated R887 INCENP antibody, and amounts of loading proteins were evaluated by staining with MemCode^TM^ Reversible Protein Stain. **C.** 293T cells were co-transfected with a FLAG-INCENP-WT (amino acids 821-918) vector or a FLAG-INCENP-R887A (amino acids 821-918) vector and an HA-PRMT1 vector. The samples were immunoblotted with anti-mono-methylated R887 INCENP, anti-FLAG and anti-AURKB antibodies after immunoprecipitating with anti-FLAG M2 agarose (Sigma-Aldrich). **D.** HeLa cells were transfected with FLAG-PRMT1-WT and immunocytochemistry was performed with anti-FLAG (Sigma-Aldrich, M2, dilution: 1:100, green) and anti-R887meINCENP (dilution: 1:100, green) antibodies. Nuclei were stained with 4′,6-diamidino-2-phenylindole (DAPI, blue). Transfected HeLa cells exhibited higher INCENP and R887meINCENP integrated densities.

We performed an *in vitro* methyltransferase assay using recombinant PRMT1 protein with a variety of recombinant non-histone proteins that are involved in human tumorigenesis, such as INCENP, AURKB, PTEN, ERK1, HRAS and β-catenin, to identify a novel substrate of PRMT1 in human cancer, and found that PRMT1 methylates INCENP (Figure 1A). To further characterize a methylation site(s) of INCENP by PRMT1, we applied liquid chromatography-tandem mass spectrometry (LC-MS/MS) analysis and identified arginine 887 (R887) to be mono-methylated (Figure 1B–1D). Subsequently we generated an anti-R887 mono-methylated INCENP antibody with high specificity as described in “Methods” section; the high affinity against the methylated peptide was confirmed by enzyme-linked immunosolvent assay (ELISA) (Figure 2A). To further validate the specificity of this antibody, we conducted an *in vitro* methyltransferase assay, followed by western blot analysis using the methylation-specific antibody. As shown in Figure 2B, the antibody specifically recognized R887-methylated INCENP protein after incubation with PRMT1 while no band was detected in the absence of PRMT1. In addition, 293T cells were co-transfected with a FLAG-INCENP-WT vector or a FLAG-INCENP-R887A vector and an HA-PRMT1 vector, and western blot analysis was performed after immunoprcipitation of the INCENP protein (Figure 2C). The methylation-specific signal was detected in wild-type INCENP but not in R887-substituted INCENP (INCENP-R887A), further confirming the specificity of the methylation specific antibody. Interestingly, the binding affinity of R887A-substituted INCENP to AURKB was much lower than that of wild-type INCENP, indicating the interaction between INCENP and AURKB was significantly affected when R887 was substituted (Figure 2C). This result may imply that the R887 methylation of INCENP is likely to be critical for the interaction with AURKB. Furthermore, we found R887-methylation levels in HeLa cells were enhanced when PRMT1 was introduced (Figure 2D). Taken together, these results demonstrate that PRMT1 methylates INCENP at arginine 887 both *in vitro* and *in vivo*.

### PRMT1-mediated R887 methylation is critical for AURKB activation

**Figure 3:**
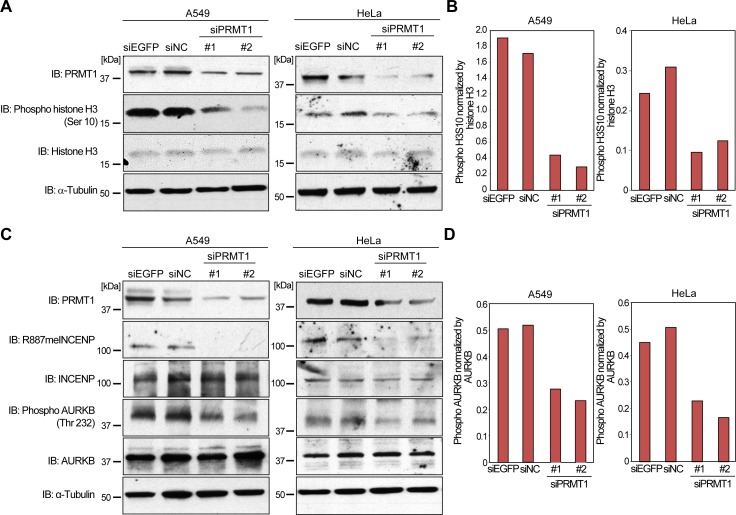
Regulation of AURKB activity by PRMT1 **A.** Effects of PRMT1 knockdown on the phosphorylation of histone H3 at serine 10. A549 and HeLa cells were transfected with control siRNAs (siEGFP and siNC) and PRMT1 siRNAs (#1 and #2) for 72 h. Cells were lysed with CelLytic^TM^ M reagent, and samples were immunoblotted with anti-PRMT1, anti-phospho histone H3S10, anti-histone H3 and anti-α-Tubulin antibodies. **B.** X-ray films in **A.** were scanned with GS-800^TM^ calibrated densitometer (Bio-Rad), and the intensity of phospho H3S10 levels was normalized by histone H3 expression levels. **C.** PRMT1 regulates AURKB activity. A549 and HeLa cells were transfected with control siRNAs (siEGFP and siNC) and PRMT1 siRNAs (#1 and #2) for 72 h. Cells were lysed with CelLytic^TM^ M reagent, and samples were immunoblotted with anti-PRMT1, anti-R887meINCENP, anti-INCENP, anti-phospho AURKB (threonine 232), anti-AURKB and anti-α-Tubulin antibodies. **D.** X-ray films in **C.** were scanned with GS-800^TM^ calibrated densitometer (Bio-Rad), and the intensity of phospho AURKB at threonine 232 levels was normalized by AURKB expression levels.

Since R887 of INCENP is located in the IN-box domain that is required for the binding to AURKB [10, 17], this modification possibly affects the binding affinity of INCENP to AURKB. Furthermore, the association of INCENP and AURKB is considered to be important for the enzymatic activation of AURKB kinase [11, 12]. Hence, we hypothesized that PRMT1-mediated methylation of INCENP may be pivotal for AURKB activation. To evaluate this hypothesis, we knocked down PRMT1 expression in A549 human non-small cell lung cancer cells that express high levels of PRMT1 (Supplementary Figure S1), and examined phosphorylation levels of histone H3 at serine 10, which is known as a substrate of AURKB. As shown in Figure 3A and 3B, knockdown of PRMT1 clearly diminished phosphorylation levels of histone H3 at serine 10 in A549 and HeLa cells. We also conducted knockdown of PRMT1 in A549 and HeLa cells to examine phosphorylation levels of AURKB at threonine 232, which is an indicator of AURKB activity (Figure 3C and 3D). Consistently, PRMT1 knockdown also reduced phosphorylation levels of threonine 232. These results indicate that PRMT1-mediated INCENP methylation appears to be pivotal for AURKB activation.

### INCENP methylation is important for proper cell division and growth of cancer cells

**Figure 4:**
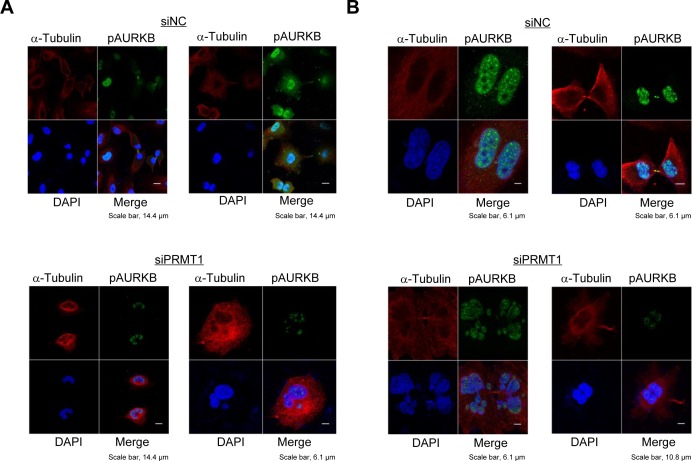
PRMT1 knockdown causes abnormal chromosome alignment and chromosome segregation A549 cells **A.** and HeLa cells **B.** were transfected with siNC (control) or siPRMT1#1 for 48 h, and then treated with 7.5 μg/ml of aphidicolin for 24 h. Cells were fixed with 4% paraformaldehyde, and then stained with an anti-phospho AURKB antibody (Alexa Fluor^®^ 488, green), an anti-α-Tubulin antibody (Alexa Fluor^®^ 594 [red]) and 4′,6′-diamidine-2′-phenylindole dihydrochloride (DAPI [blue]) 15 h after release of cell cycle. The percentage of cells, which showed abnormal chromosome alignment and chromosome segregation, were 74.4% (A549) and 72.9% (HeLa) to the total number of cells.

It is known that activated AURKB plays an important role in the proper cell division [18]. Since our data revealed that PRMT1-mediated INCENP methylation is critical for the activation of AURKB, we then examined the effect of PRMT1 knockdown on the cell division of cancer cells. Knockdown of PRMT1 resulted in the attenuation of phosphorylated AURKB, and abnormal chromosomal alignment and segregation in A549 cells (Figure 4A); a similar result was also observed when it was knocked down in HeLa cells, which overexpressed PRMT1 (Figure 4B and Supplementary Figure S1). Moreover, the phenotype of cancer cells after knockdown of PRMT1 is similar to that after knockdown of INCENP (Supplementary Figure S2). These results imply that PRMT1-mediated INCENP methylation appears to be required for the proper cell division of cancer cells.

**Figure 5:**
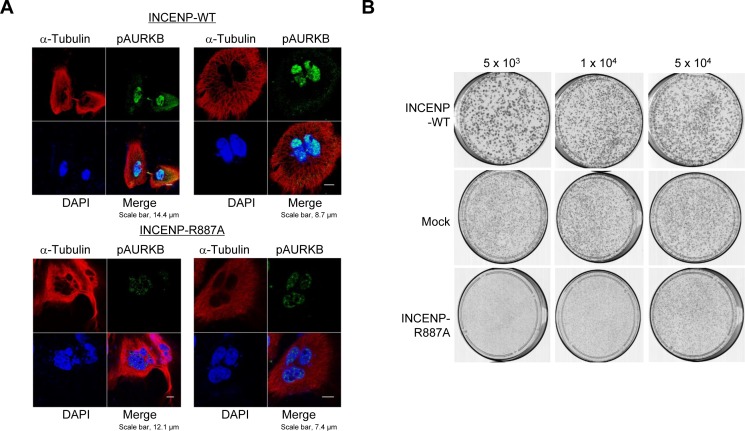
PRMT1-mediated methylation of INCENP at R887 is critical for proper mitotic progression of cancer cells **A.** HeLa cells were transfected with wild-type INCENP (INCENP-WT) and R887-subtituted INCENP (INCENP-R887A), and then stained with an anti-phospho AURKB antibody (Alexa Fluor^®^ 488, green), an anti-α-Tubulin antibody (Alexa Fluor^®^ 594 [red]) and 4′,6′-diamidine-2′-phenylindole dihydrochloride (DAPI [blue]) 15 h after release of cell cycle. The percentage of cells, which showed abnormal chromosome alignment and chromosome segregation, were 55.8% to the total number of cells. **B.** HeLa cells were transfected with pCAGGS-n3FC-Mock, pCAGGS-n3FC-INCENP-WT and pCAGGS-n3FC-INCENP-R887A for 24 h, and then re-inoculated at the concentration of 5 × 10^3^ cells/dish, 1 × 10^4^ cells/dish or 5 × 10^4^ cells/dish with the medium containing 0.8 (mg/ml) of Geneticin^®^. Cells were fixed with methanol and then stained with Giemsa.

In order to further verify the importance of INCENP methylation at R887 by PRMT1 for cell division, we overexpressed wild-type INCENP or R887A-substituted INCENP in HeLa cells and performed immunocyctochemical analysis (Figure 5A). We observed the diminishment of phospho-AURKB and an increase of multiple nuclei and/or micronuclei after overexpression of R887A-substituted INCENP. Clonogenicity assays using the same constructs and cells revealed that INCENP-R887A overexpressing cells showed slower growth rate than INCENP-WT overexpressing or Mock cells (Figure 5B and Supplementary Figure S3). The data indicate that INCENP methylation at R887 by PRMT1 is critical for the growth of cancer cells.

## DISCUSSION

We have demonstrated that the protein arginine methyltransferase PRMT1 methylates arginine 887 of INCENP, and that this methylation is critically important for the proper chromosomal segregation and cell division of cancer cells. Importantly, arginine 887 is located in the IN-box domain, which is known to bind and activate AURKB [5, 6]. Among various species, arginine 887 is well-conserved (Supplementary Figure S4) and close to a C-terminal TSS (threonine-serine-serine) motif that includes two conserved serine residues phosphorylated by AURKB [17]. This TSS phosphorylation further activates AURKB in a positive feedback loop [12, 19], which implies that methylation of INCENP at R887 by PRMT1 is likely to be a trigger of AURKB activation. Our data clearly indicate that methylation of arginine 887 is critical for the interaction with AURKB (Figure 2C). Taken together, PRMT1-mediated INCENP methylation at arginine 887 appears to be a key regulator of AURKB activation. Unfortunately, since X-ray crystal structure of the portion including R887 and the TSS motif in INCENP protein is not available at present, we are unable to verify this hypothesis by the three dimensional structural analysis.

It should be noted that aurora kinase is widely recognized as a therapeutic target of cancer [20]. AURKB has been extensively investigated as a potential target in leukemia after it was discovered that AURKB was overexpressed in leukemia cells of acute lymphoblastic leukemia, acute myeloid leukemia and chronic lymphoid leukemia [20–23]. Subsequently, dysregulation of AURKB was reported in various types of solid tumor, and clinical trials of anti-cancer drugs targeting AURKB have been actively conducted [24–27]. Our current study clearly revealed that PRMT1-mediated INCENP methylation is a critical event for activation of AURKB, and attenuation of this methylation led to repression of the AURKB enzyme activity in cancer cells, implying that inhibition of INCENP methylation by PRMT1 appears to be a good approach to develop anti-cancer drugs. Indeed, transactivation of PRMT1 was found in various types of cancer [16, 28–31], and inhibition of PRMT1 significantly suppresses growth of cancer cells [16, 30, 31]. Of these signatures, PRMT1 is considered as an ideal target for anti-cancer therapy. Although any clinical study has not been started yet, small chemical compounds targeting protein arginine methyltransferases including PRMT1 have been developed as anti-tumor agents [32–34].

Accumulated evidence has indicated that methylation of arginine and lysine residues plays an important role in the regulation of many biological processes like phosphorylation does, and its deregulation is almost certainly involved in human tumorigenesis [14, 35–37]. Further detailed analyses of protein methylation should explore the fundamental role of this post-translational modification in human cancer and the importance of protein methyltransferase as a target of development of novel anti-cancer treatment.

## MATERIALS AND METHODS

### Antibodies

The following primary antibodies were used: anti-FLAG (mouse, M2; Sigma-Aldrich, St. Louis, MO; dilution used in ICC: 1:100), anti-FLAG (rabbit, F-7425; Sigma-Aldrich; dilution used in WB: 1:2000), anti-PRMT1 (NQ-15) (rabbit, P6871; Sigma-Aldrich; dilution used in WB: 1:500), anti-AURKB (rabbit, #3094; Cell Signaling Technology; dilution used in WB: 1:500), anti-phospho AURKB (T232) (rabbit, ab115793; Abcam; dilution used in WB: 1:500), anti-INCNEP (rabbit, ab12183; Abcam; dilution used in WB: 1:500), anti-α-Tubulin (mouse, DM1A; CALBIOCHEM, Billerica, MA; dilution used in WB: 1:1000), anti-phospho histone H3 (serine 10) (rabbit, #9701S, Cell Signaling Technology, dilution used in WB: 1:500) and anti-histone H3 (rabbit, ab1791; Abcam; dilution used in WB: 1:500). An anti-887 mono-methylated INCENP antibody (Sigma-Aldrich; dilution used in WB: 1:500, ICC: 1:100) was produced in rabbit immunized with a synthetic peptide.

### Cell culture

HeLa, 293T and A549 cell lines were obtained from American Type Culture Collection (ATCC), and detailed information of DNA profile is described in Supplementary Table S1. HeLa, 293T and A549 cells were grown in the monolayers in appropriate media supplemented with 10% fetal bovine serum and 1% antibiobic/antimycotic solution (Sigma-Aldrich): Dulbecco's modified Eagle's medium (D-MEM) for 293T cells; Eagle's minimal essential medium (E-MEM) for HeLa cells; RPMI-1640 for A549 cells. All cells were maintained at 37°C in humid air with 5% CO_2_ condition. Cells were transfected with FuGENE HD (Promega, Fitchburg, WI) according to manufacturer's protocols [38].

### *In vitro* methyltransferase assay

*In vitro* methyltransferase assays were described previously [37, 39–46]. Briefly, recombinant GST-tagged INCENP (amino acids 821-918) proteins were incubated with recombinant PRMT1 and 2 μCi S-adenosyl-L-[methyl-^3^H]-methionine (SAM) (Perkin Elmer, Waltham, MA) in a mixture of methylase activity buffer (50 mM Tris-HCl at pH 8.8, 10 mM DTT and 10 mM MgCl_2_) for 2 h at 30°C. After denaturing, samples were separated by SDS-PAGE, blotted to PVDF membrane and visualized by MemCode^TM^ Reversible Stain (Thermo Fisher Scientific, Waltham, MA) and fluorography.

### Mass spectrometry

Mass spectrometry was described previously [43]. INCENP samples treated with BSA or PRMT1 were separated on SDS-PAGE and stained with Simply Blue Safe Stain (Life Technologies). The INCENP bands were excised and digested in gel with trypsin. Then digest was analyzed by nano liquid chromatography–tandem mass spectrometry (LC-MS/MS) using Q Exactive mass spectrometer (Thermo Fisher Scientific). The digests were applied to a nano ESI spray column (75 μm [ID] × 100 mm [L], NTCC analytical column C18, 3 μm, Nikkyo Technos, Tokyo, Japan) and eluted with a linear gradient of 0%–35% buffer B (100% ACN and 0.1% formic acid) at a flow rate of 300 nL/min over 10 min (Easy nLC; Thermo Fisher Scientific). The eluates were analyzed on line to the mass spectrometer, and the MS and MS/MS spectra were obtained using a data-dependent TOP5 method in a positive mode. The MS/MS spectra were searched against the in-house database using local MASCOT server (version 2.3; Matrix Sciences, London, United Kingdom).

### Immunocytochemistry

Cells were fixed in 4% paraformaldehyde in 0.1M phosphate buffer at 4°C for 1 h, permeabilized in 0.1% Triton X-100 (Sigma-Aldrich) for 3 min at room temperature and blocked with 3% BSA for 1 h at room temperature. Fixed cells were incubated with anti-FLAG, anti-R887 methylated INCENP, anti-α-Tubulin and anti-phospho-AURKB (T232) antibodies overnight at 4°C, followed by incubation with Alexa Fluor-conjugated secondary antibody (Thermo Fisher Scientific) and observed using Leica confocal microscopy (SP5 tandem Scanner Spectral 2-Photon Confocal).

### Western blot

Samples were prepared from the cells lysed with CelLytic^TM^ M lysis reagent (Sigma-Aldrich) supplemented with complete protease inhibitor cocktail (Roche Applied Science, Penzberg, Germany). Whole cell lysates or immunoprecipitated samples were separated by SDS-PAGE and blotted to nitrocellurose membrane. Protein bands were detected by incubating with horseradish peroxidase (HRP)-conjugated antibodies (GE Healthcare, Little Chalfont, UK) and visualizing with enhanced chemiluminescence (GE Healthcare).

### Small interfering RNA transfection

siRNA oligonucleotide duplexes were purchased from Sigma-Aldrich for targeting PRMT1 transcripts. siEGFP and siNegative control (siNC), which is a mixture of three different oligonucleotide duplexes were used as control siRNAs [16, 47–49]. The siRNA sequences are described in Supplementary Table 2. siRNA duplexes were transfected with Lipofectamine RNAi max (Thermo Fisher Scientific).

### Immunoprecipitation

Transfected 293T cells were lysed with CelLytic^TM^ M cell lysis reagent (Sigma-Aldrich) containing a complete protease inhibitor cocktail (Roche Applied Science). In a typical immunoprecipitation reaction, 300 μg of whole-cell extract was incubated with an optimum concentration of primary antibody. After the beads had been washed 3 times in 1 ml of TBS buffer (pH 7.6), proteins that bound to the beads were eluted by boiling in Lane Marker Reducing Sample Buffer (Thermo Fisher Scientific).

### Cloning of expression vectors

Full-length of PRMT1 and C-terminal portion of INCENP (amino acids 821-918) were sub-cloned into the pCAGGS-n3FC vector. Full-length PRMT1 was also sub-cloned into the pCAGGS-nHC vector. Each expression vector was transfected into 293T cells and HeLa cells using FuGENE HD transfection reagents.

## SUPPLEMENTARY MATERIAL FIGURES AND TABLES


