# Supplementary material for: PRMT1 promotes mitosis of cancer cells through arginine methylation of INCENP
Source: Oncotarget. 2015 Oct 9;6(34):35173–82. doi: 10.18632/oncotarget.6050 (PMC4742097; doi:10.18632/oncotarget.6050)
Supplement: Supplementary file 1 [file oncotarget-06-35173-s001.pdf]

PRMT1 promotes mitosis of cancer cells through arginine methylation of INCENP

Supplementary Material

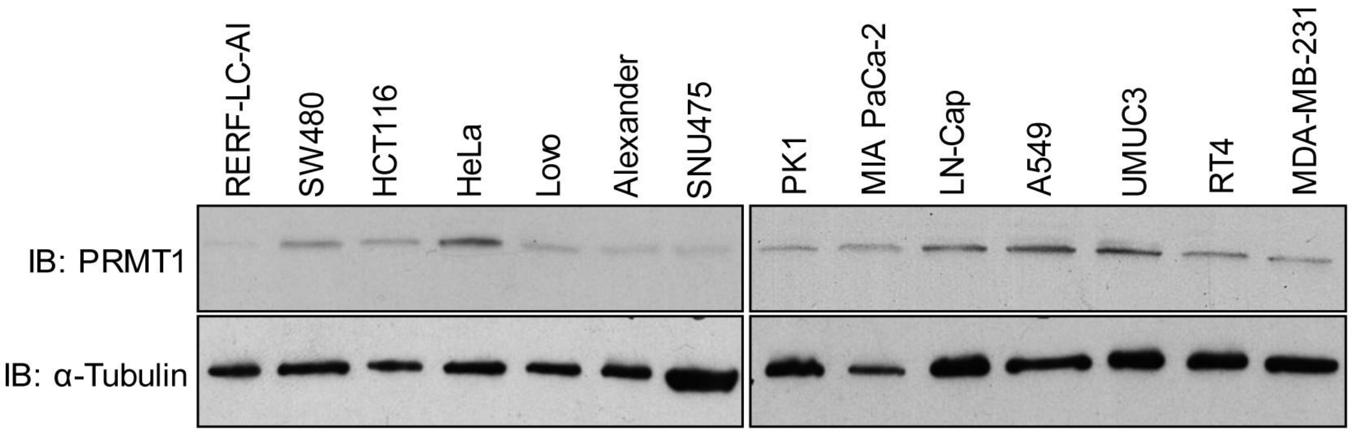

Figure S1: Protein expression levels of PRMT1 in various types of cancer cell lines. Fourteen cancer cell lines were lysed with CelLytic M reagent and samples were immunoblotted with an anti-PRMT1 antibody. α-Tubulin protein expression was used as an internal control.

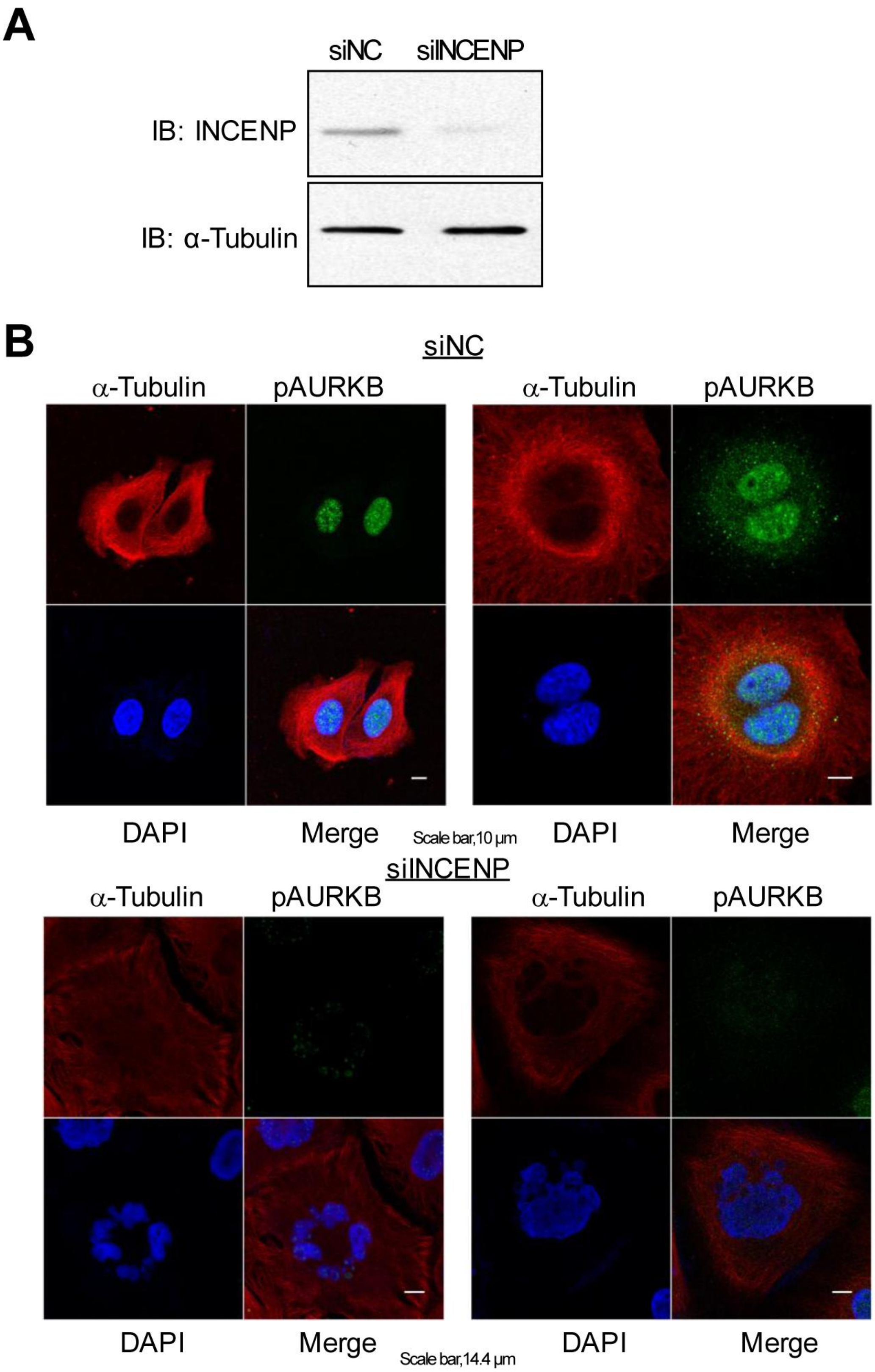

Figure S2: Knockdown of INCENP results in abnormal chromosome alignment and segregation. (A) HeLa cells were transfected with siNC and siINCENP for 72 h. Samples were immunoblotted with anti-INCENP and anti-α-Tubulin antibodies. (B) HeLa cells were transfected with siNC and siINCENP for 72 h. Cells were fixed with 4% paraformaldehyde, and stained with anti-α-Tubulin (Alexa Fluor® 594 [red]), anti-phospho AURKB antibodies, and 4',6'-diamidine-2'-phenylindole dihydrochloride (DAPI [blue]).

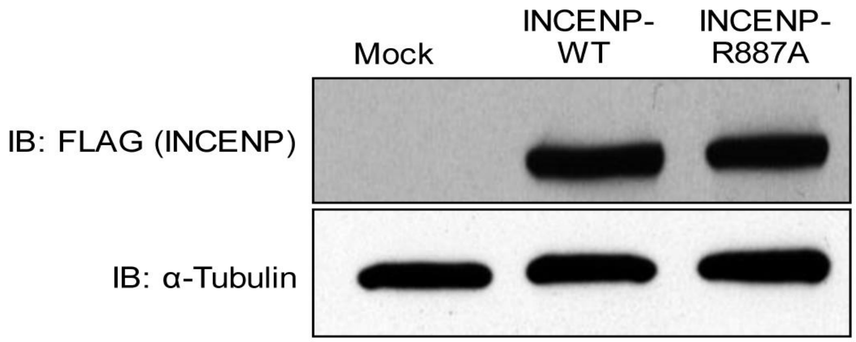

Figure S3: Expression check of FLAG-INCENP-WT and FLAG- INCENP-R887A in HeLa cells in Figure 5B. Samples were immunoblotted with anti-FLAG and anti-α-Tubulin antibodies.

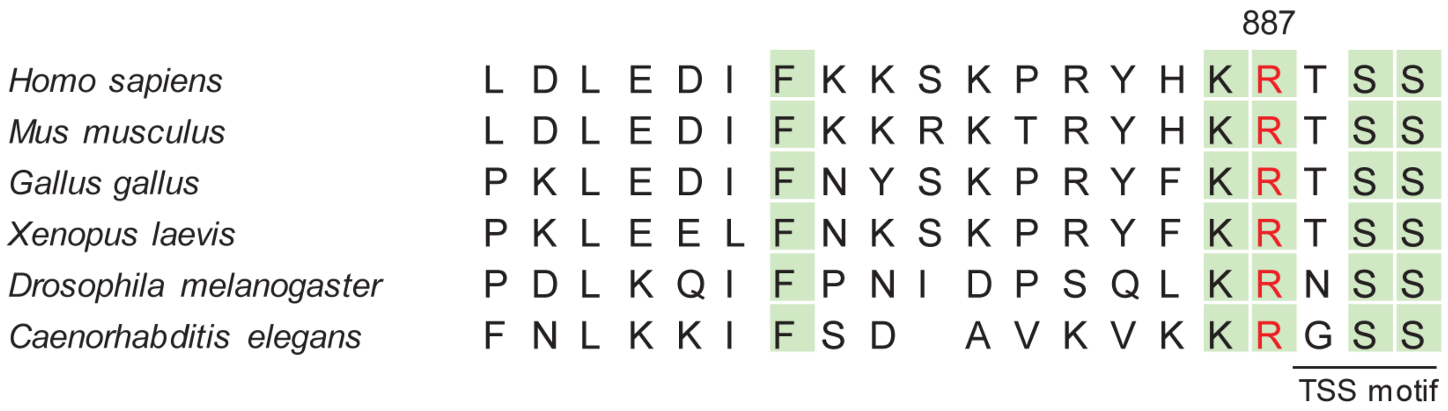

Figure S4: Amino acid sequence alignment of the IN-domain of INCENP. R887 is conserved among various species. Green color highlights the amino acid that is conserved from *Caenorhabditis elegans* to *Homo sapiens*.

Table S1: Information of certificated cell lines

Name	Certification institution	Tested method	DNA profile
**293T**	ATCC	STR	Amelogenin: X CSF1PO: 11, 12 D13S317: 12, 14 D16S539: 9, 13 D5S818: 8, 9 D7S820: 11 THO1: 7, 9.3 TPOX: 11 vWA: 16, 18, 19
**HeLa**	ATCC	STR	Amelogenin: X,Y CSF1PO: 11,12 D13S317: 11,14 D16S539: 9,11 D5S818: 11,12 D7S820: 10,11 THO1: 8 TPOX: 8 vWA: 15
**A549**	ATCC	STR	Amelogenin: X,Y CSF1PO: 10,12 D13S317: 11 D16S539: 11,12 D5S818: 11 D7S820: 8,11 THO1: 8,9.3 TPOX: 8,11 vWA: 14

ATCC; American Type Culture Collection

Table S2: siRNA sequences

siRNA name		Sequence	
siEGFP		Sense	GCAGCACGACUUCUUCAAG
		Antisense	CUUGAAGAAGUCGUGCUGC
siNegative control (cocktail)	Target #1	Sense	AUCCGCGCGAUAGUACGUA
		Antisense	UACGUACUAUCGCGCGGAU
	Target #2	Sense	UUACGCGUAGCGUAAUACG
		Antisense	CGUAUUACGCUACGCGUAA
	Target #3	Sense	UAUUCGCGCGUAUAGCGGU
		Antisense	ACCGCUAUACGCGCGAAUA
siPRMT1#1		Sense	GAGUUCACACGCUGCCACA
		Antisense	UGUGGCAGCGUGUGAACUC
siPRMT1#2		Sense	GCCACAAGAGGACCGGCUU
		Antisense	AAGCCGGUCCUCUUGUGGC
siINCENP		Sense	CUCAGAAGAACCGACGGAA
		Antisense	UUCCGUCGGUUCUUCUGAG
